# Reducing Calibration Time in PET Systems Based on Monolithic Crystals

**DOI:** 10.3389/fmed.2021.734476

**Published:** 2021-11-10

**Authors:** Marta Freire, Gabriel Cañizares, Sara Echegoyen, Andrea Gonzalez-Montoro, Antonio J. Gonzalez

**Affiliations:** Instituto de Instrumentación para Imagen Molecular, Centro Mixto CSIC—Universitat Politècnica de València, Valencia, Spain

**Keywords:** positron emission tomography, monolithic crystals, calibration, total-body PET, whole-body PET

## Abstract

In the past years, the gamma-ray detector designs based on the monolithic crystals have demonstrated to be excellent candidates for the design of high-performance PET systems. The monolithic crystals allow to achieve the intrinsic detector resolutions well below state-of-the-art; to increase packing fraction thus, increasing the system sensitivity; and to improve lesion detectability at the edges of the scanner field of view (FOV) because of their intrinsic depth of interaction (DOI) capabilities. The bottleneck to translate to the clinical PET systems based on a large number of monolithic detectors is eventually the requirement of mechanically complex and time-consuming calibration processes. To mitigate this drawback, several methods have been already proposed, such as using non-physically collimated radioactive sources or implementing the neuronal networks (NN) algorithms trained with simulated data. In this work, we aimed to simplify and fasten a calibration process of the monolithic based systems. The *Normal* procedure consists of individually acquiring a 11 × 11 ^22^Na source array for all the detectors composing the PET system and obtaining the calibration map for each module using a method based on the Voronoi diagrams. Two reducing time methodologies are presented: (i) *TEST1*, where the calibration map of one detector is estimated and shared among all others, and (ii) *TEST2*, where the calibration map is slightly modified for each module as a function of their detector uniformity map. The experimental data from a dedicated prostate PET system was used to compare the standard calibration procedure with both the proposed methods. A greater similarity was exhibited between the *TEST2* methodology and the *Normal* procedure; obtaining spatial resolution variances within 0.1 mm error bars and count rate deviations as small as 0.2%. Moreover, the negligible reconstructed image differences (13% deviation at most in the contrast-to-noise ratio) and almost identical contrast values were reported. Therefore, this proposed method allows us to calibrate the PET systems based on the monolithic crystals reducing the calibration time by approximately 80% compared with the *Normal* procedure.

## Introduction

In the PET detectors, two main types of scintillator crystals are usually employed namely, pixelated and monolithic. The advantages and disadvantages of each one are extensively described elsewhere (1). They offer intrinsic resolutions that are well below the state-of-the-art and an improvement of the system sensitivity, as they do not contain zero detection zones, unlike the pixelated crystals. But the most significant feature of monolithic crystals is their inherent access to the light distribution (LD) profile of the scintillation events which allows to retrieve, in addition to the planar impact coordinates (*x,y*), accurate photon depth of interaction (DOI) information, unlike the pixelated crystals that require additional components to provide 3D positioning information (2, 3). The DOI information permits to correct for the parallax errors, which strongly affect the systems with small apertures (i.e., small animal and organ dedicated scanners), but also at the edges of the field of view (FOV) in the human size scanners. Both width and position of the source profile improve when applying the DOI correction independently of the system diameter (4, 5). Recently, the monolithic crystals are employed in the PET scanners achieving high sensitivity and spatial resolution (6–8). Moreover, regarding cost, analyzing the different providers for scintillator crystals and studying the price differences between the several pixel arrays and monolithic crystals with similar volumes, it can be concluded that they are cheaper than the traditional pixelated scintillators for the pixel sizes smaller than 1.5 mm × 1.5 mm, as the ones used in the pre-clinical PET imaging.

To accurately determine the energy and 3D impact position in the monolithic-based PET detectors, the calibration processes accounting for the possible non-uniformities or edge effects are required (9). The non-uniformities arise from different gains in the photosensors or readout channels, and eventually by the crystal light yields abnormalities. The edge effects result from the scintillation light truncation toward the crystal edges, reducing the accuracy of the photon impact coordinates determination and energy discrimination. For the pixelated-based detectors, the flood maps are easily and quickly found, since one source can be placed at the center of the PET scanner providing information of all the pixel elements. However, for the monolithic-based detectors, the calibration processes are typically based on scanning a collimated small size source across the entire monolithic surface while recording the measured and mechanical/known source positions (1). This procedure must be applied for each detector module of the PET scanner, which results in the time-consuming calibrations and requires using entangled hardware set-ups (9). For one single detector, the measurement for obtaining reference data might last about 30 min even when using the high activity sources.

Multiple methods have been proposed to ease the calibration processes in the monolithic assemblies; such as using reference data corresponding to a line of irradiation points instead of singular points (10 −13), utilizing an array of collimated sources (10), or using non-physically collimated sources (11, 12). An alternative approach, not requiring the calibration for each detector block of the PET system, is to carry out an accurate simulation of the detector responses either for Neural Networks (NN) training (13, 14) or for the generation of look-up-tables (LUTs) to be applied using the maximum likelihood expectation maximization methods (MLEM) (15).

In this work, we propose an approach to apply the detector calibration process based on the Voronoi diagrams (10) in the PET scanners based on a large number of monolithic detectors. The proposed methodology significantly reduces the calibration times while accounts and corrects for the possible differences among each individual detector module. Shortly, the method suggests using the combined accurate calibration of few detectors, to be applied after some tuning provided by uniform radiation, to all the other detectors. In the following, we describe this rather simple methodology, but never studied before in detail, and its experimental validation employing data from a prostate dedicated clinical PET scanner (16).

## Materials and Methods

### Materials

**Figure 1:**
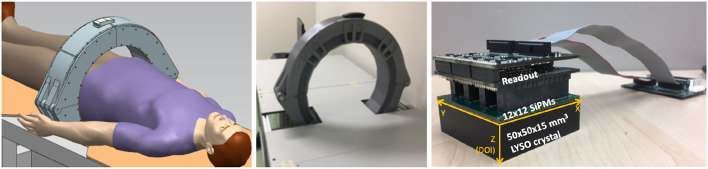
The sketch (left) and photograph (right) of the prostate dedicated PET system used during the calibration tests.

Data were experimentally acquired using a clinical PET specifically designed for prostate imaging. The scanner is composed of a single ring with 24 detectors (16), each one comprising a LYSO:Ce (Lu_1.8_Y_2_SiO_5_:Ce) monolithic crystal of 50 × 50 × 15 mm with the lateral surfaces black painted (absorbent paint) and the entrance face, such as a retro-reflector layer (10, 17), as shown in the images of the system in Figure 1. Each scintillation crystal is coupled to a photosensor array of 12 × 12 silicon photomultipliers (SiPMs) with 3 × 3 mm active area and 4.2 mm pitch (52% active are coverage) by means of optical grease (BC-630, Saint Gobain, France). The readout scheme provides the row and column SiPM signals, thus allowing to determine the 3D photon impact coordinates within the crystal (4, 18). The detector output signals are fed into a data acquisition (DAQ) system based on the 12-bit analog-to-digital converters (ADCs) with 1 GB ethernet connection, and the summed signal of either all SiPM rows or columns, was fed into a trigger board that allows coincidences within a 5 ns coincidence window. Further details about the system can be found in the reference (16).

The planar impact coordinates (*x, y*) were calculated using the rows and column SiPM signals by applying a modified version of the center of gravity algorithm (COG) in which the row and column values are risen to the power of 2 to improve the system linearity (19). The DOI value was estimated as *E/I*_*max*_where E is the energy calculated as the sum of the rows or columns, and *I*_*max*_ is the maximum value of the row or column, respectively (12).

### Calibration Process

**Figure 2:**
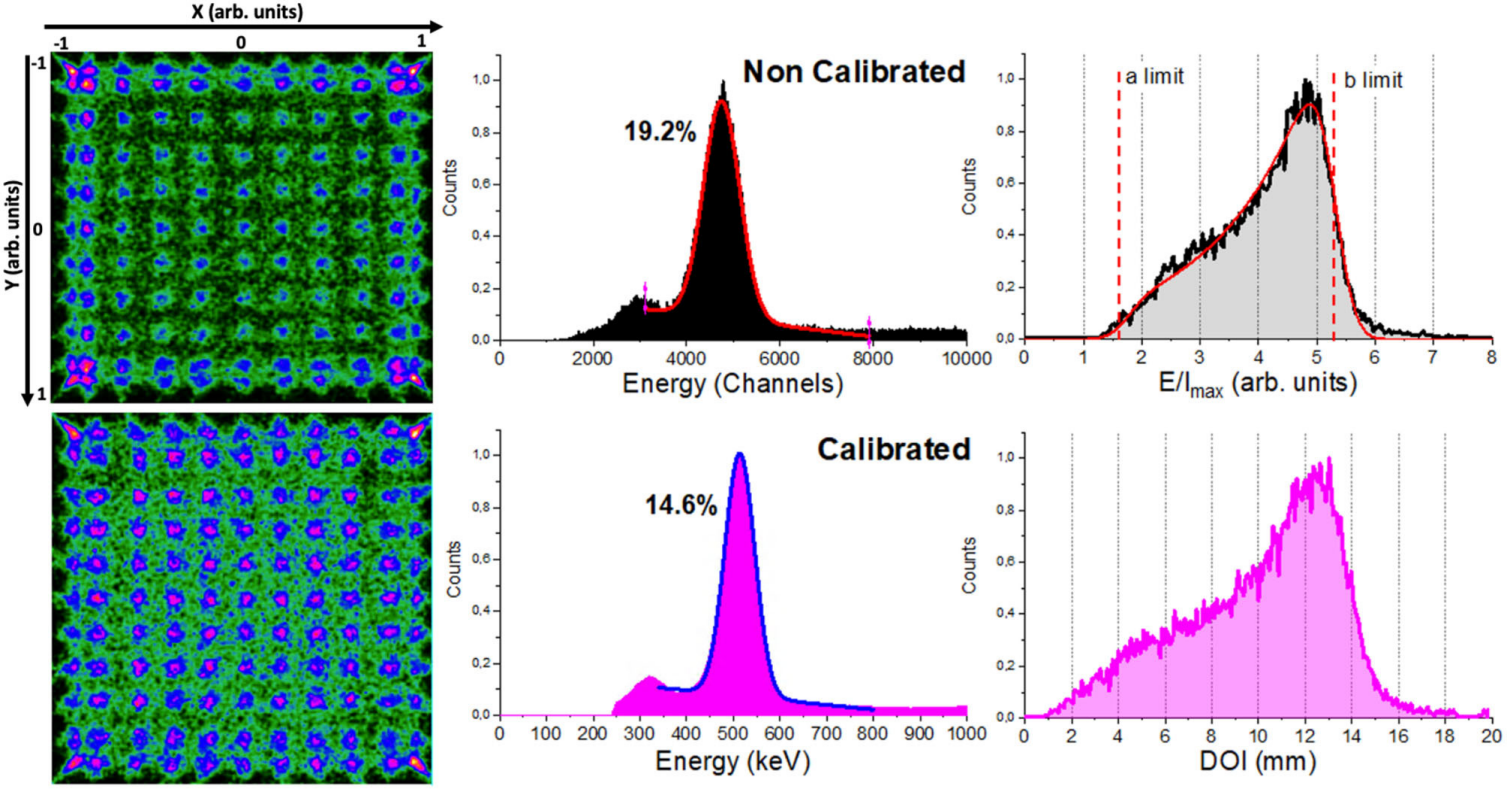
From left to right, the image flood maps of the 11 × 11 ^22^Na collimated sources before (top) and after (bottom) calibration using the *Normal* method, energy spectra, and depth of interaction (DOI) distribution for the whole detector.

Instead of sequentially moving individual radioactive sources across the crystal surface, which requires long calibration times, we used an array of 11 × 11 ^22^Na radioactive sources (4.6 mm pitch and 1 mm in diameter, total activity ~10 μCi) placed at the known positions. A 30 mm thick tungsten collimator, with drilled holes of 1.2 mm in diameter, was accurately aligned with the sources and placed at each crystal entrance. The acquired reference data were later post-processed using a software collimation method (defined as a trade-off between the statistics and spatial resolution) that rejects the lines of response (LORs) with angles larger than 1.2 degrees measured from the detector normal (2). These two-steps, acquisition and collimation, resulted in the accurate flood maps composed by 121 measured positions as those shown in Figure 2 (left).

The calculated 3D photon impact position and energy were calibrated using a method based on the Voronoi diagrams. The flood map of the 11 × 11 ^22^Na sources (as shown in Figure 2) is used to generate a Voronoi diagram, thus permitting the partition of the crystal surface into 121 Voronoi cells and the extraction of five Voronoi factors for each cell (10). The VoronoiFactor_X_ and VoronoiFactor_Y_ were calculated as the deviation of the measured source position to the mechanical position and the VoronoiFactor_E_ was determined as the deviation of the energy photopeak value in the channels to the value corresponding to the central Voronoi cell. Finally, we determined the lower and upper limits (*a* and *b* parameters) and sigma (σ_int_) of the E/I_max_ histogram for each Voronoi region using the DOI analytical expression extracted from the reference (14) (as shown in Figure 2). Two Voronoi factors were calculated corresponding to the limits *a*-σ_int_ and *b*+σ_int_ and then, considered to be equal to 0 and 15 mm (crystal thickness) to calibrate the measured E/I_max_ into millimeters. As shown in the reference (10) for more detail of the process. These Voronoi factors were used to obtain five LUTs: two corresponding to the planar XY coordinates {LUT_X_, LUT_Y_}, two to the DOI {LUT_DOI1_, LUT_DOI2_}, and another one corresponding to the energy {LUT_Energy_}. These LUTs are finally used to calibrate every impact. Data from the subjects or phantoms are off-line calibrated applying the calculated LUTs in an event-by-event process that includes a correction to the true LOR (parallax error compensation).

**Figure 3:**
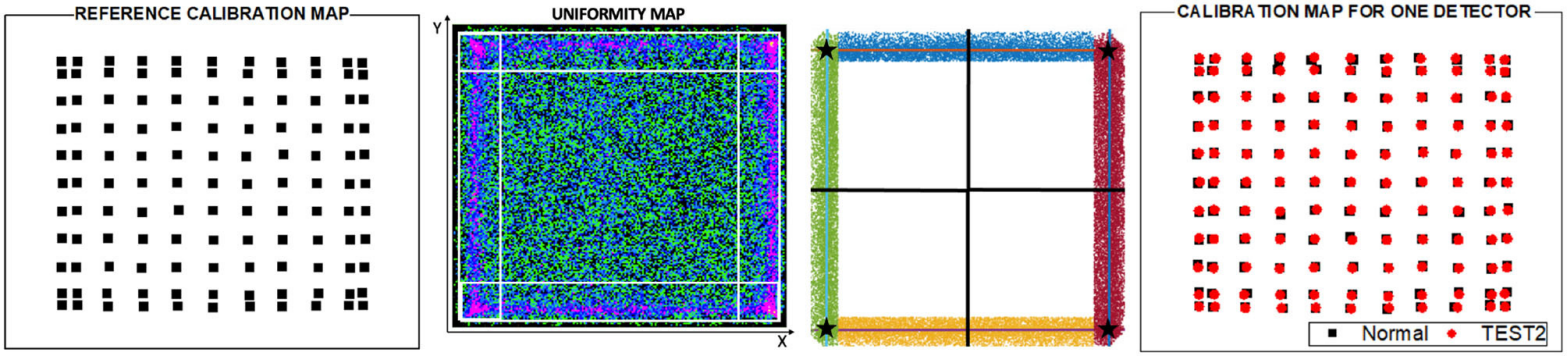
From left to right, a reference calibration map obtained as the average of the calibration positions of three random detectors, example of a detector uniform map for one detector used to obtain the calibration map in *TEST2*, surface partition obtained from the four corners calculated as the intersection of the lines following the event accumulation and calibration positions obtained for one detector in the Normal calibration and after applying the TEST2.

We have tested three different calibration methods, a conventional one detector-by-detector calibration, and two proposed modifications to shorten the calibration times:

- *Normal*, the 24 detectors of the PET scanner were individually calibrated as described above. This means, that a set of 5 individuals {LUT_X, Y, DOI1, DOI2, Energy_} is generated from the flood map of each detector module. This calibration is considered as the ground-truth for comparison purposes. Figure 2 shows the flood map of the 11 × 11 ^22^Na sources, the energy and DOI histograms for one detector module of the prostate dedicated PET before (top panels) and after (bottom panels) calibration. Acquisition using the described array and activity might last about 2–3 h per detector, thus 48–72 h for the whole system without stop (at least 6 working days). Notice that the higher activities and the use of non-encapsulated sources, such as ^18^F could accelerate these processes but potentially increase the radiation associated risk.
- *TEST1*, the calibration set of only one random detector is carried out and, therefore only its {LUT_X, Y, DOI1, DOI2, Energy_} are generated and shared among the other detectors without further corrections. With this approach, a total process calibration time of ~ 3 h for the entire scanner was required. We have evaluated this method for two random detectors: *T1* and *T1B*, corresponding to the detectors M2 and M6, respectively.
- *TEST2*, three random detectors of the PET scanner were individually calibrated and, to avoid an outlier detector performance, an averaged reference calibration map was obtained using the mean values of the calibration positions of the three detectors (as shown in Figure 3 left). Thereafter, the calibration maps for each other detector were determined applying a shift map to such reference calibration map. The shift map was generated for each detector using their uniformity maps (as shown in Figure 3) acquired placing a relatively large uniform activity phantom at the center of the scanner FOV. Event accumulation can be observed at the edges of the uniform map due to the truncation of the LD closer to the edge of the monolithic crystal. The *x* and *y* coordinates for these regions were plotted, as shown in Figure 2, and a linear fit was used to estimate the slope following that event accumulation. The intersection of the lines allowed us to calculate the coordinates of the four corners. Then, four shift factors with respect to the reference ones were calculated and a natural neighbor interpolation methodology considering the four corners was applied to obtain the shift map for the entire surface. The shift map for each module was applied to the reference calibration map to obtain the new calibration map corresponding to each detector. Finally, the calibration maps were used to determine the Voronoi factors according to the reference (20). The Voronoi factors corresponding to the DOI and energy, were calculated using the uniformity measurements. A total calibration time of ~ 10 h was consumed as: the uniformity acquisition (~1 h) plus the three detectors calibration maps (6–9 h). For this case, three sets of three different detectors were used defining: *T2, T2B*, and *T2BB*, in particular detectors [M1, M9, and M21], [M5, M18, and M24], and [M7, M15, and M20] were used, respectively.

### Evaluation of the Calibration Processes

The calibration accuracy of the proposed methods was evaluated by comparing the LUTs for *TEST1* and *TEST2* with the ground truth provided by the *Normal* case for each detector module of the prostate PET system. Thus, the correlation factors (CF) corresponding to X, Y, DOI1, DOI2, and energy, respectively, were determined for each detector module as:


(1) $$\begin{array}{l} CF_{X,Y,DOI1,DOI2,Energy}^{i}\\ =\frac{{(VoronoiFactor\;value_{X,Y,DOI1,DOI2,Energy}^{i})}_{TEST}}{{(VoronoiFactor\;value_{X,Y,DOI1,DOI2,Energy}^{i})}_{Normal}} \end{array}$$

where, *i* goes from 1 to 121 (each Voronoi diagram contains 121 values because 11 × 11 sources array was used for the calibration). Notice that, the range of values for the VoronoiFactor_X_ and VoronoiFactor_Y_ is [−1, 1] in arb. units; for the VoronoiFactor_E_ it is [0, ~10000] in channels and for the VoronoiFactor_DOI1, DOI2_, it is [1, 8] in arb. units (as shown in Figure 2 top). The mean of the 121 CF^i^ values was calculated, obtaining five *CF* values corresponding to X, Y, DOI1, DOI2, and energy for each detector module. Finally, the mean of the *CF_X,Y,DOI1,DOI2,Energy_* values of all detector modules were calculated and considered as a good estimator of the validity of the two proposed approaches.

In addition, the three calibration methods were compared using the reconstructed images from the following datasets:

- Data of a small size ^22^Na source (0.25 mm in diameter and ~ 22 μCi activity) scanned across the radial axis of the scanner. The spatial resolution was estimated as the full width at half of the maximum (FWHM) of the source profiles.
- Data acquired during the evaluation of the noise equivalent count rate (NECR) of the system. This dataset was used to provide hints about the count rates capabilities of the system as a function of the calibration method. Sub-optimal calibration of the detectors might lead to a decrease in the count rates.
- Data acquired using a custom designed image quality (IQ) phantom made out of Polymethyl methacrylate (PMMA) with an outer diameter of 135 and 103 mm height. The IQ phantom contains six capillaries with diameters of 20, 15, 12, 9, 6, and 4.5 mm and 60 mm height each placed inside a warm background. A capillaries-to-background concentration ratio of 38 was used.

The reconstruction of the acquired data was performed using the Customizable and Advanced Software for Tomographic Reconstruction (CASToR) platform (21) and the ordered subset expectation maximization (OSEM) algorithm, with voxels sizes of 1 × 1 × 1 mm and virtual detector pixels of 1 × 1 mm. During the reconstruction process, three iterations and two subsets were used when the small size sources were imaged, whereas eight iterations and two subsets were employed for the image quality phantom. Additionally, both the attenuation and normalization corrections were applied. For the attenuation correction, the transmission information of a previous CT acquisition was used. The normalization was applied using data of an annulus filled with fluorodeoxyglucose (FDG) [as shown in reference (11)] and processed using the three different calibration approaches.

We have quantitatively evaluated the reconstructed IQ phantom calculating the contrast-to-noise ratio (CNR) and the contrast for all cases as:


(2) $$\begin{array}{rcl} CNR & = & \frac{Mean\;hot\;spot\;VOI-BackGround\;level}{Background\;standard\;deviation} \end{array}$$

(3) $$\begin{array}{rcl} Contrast\;\left(\%\right) & = & 100\times \frac{Mean\;hot\;spot\;VOI-Background\;level}{Mean\;hot\;spot} \end{array}$$

where VOI stands for the Volume of Interest selected. Then, 12 VOIs were drawn distributed along the uniform warm area of the phantom to obtain the *background level* and *SD*. To calculate the *mean hot spot* values, six VOIs were defined fitting each capillary dimension but with a centered height of 25 mm.

## Results

### Detector Accuracy

**Figure 4:**
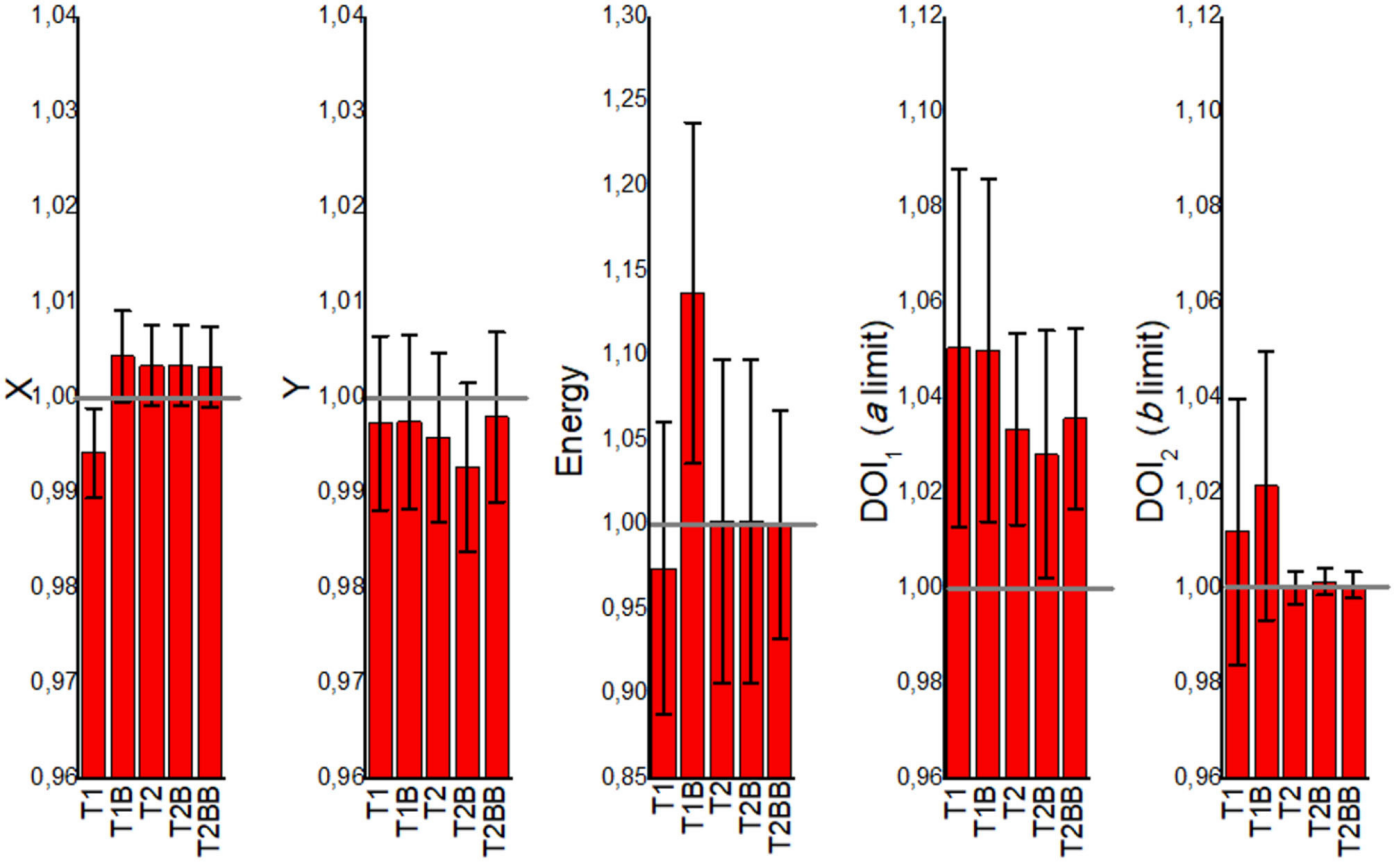
The mean correlation factor (CF^i^) values obtained from the VoronoiFactor_X_, VoronoiFactor_Y_, VoronoiFactor_E_, VoronoiFactor_DOI1_, and VoronoiFactor_DOI2_ for all the detectors and calibration positions, and for all the proposed calibration cases.

Figure 4 shows the mean values for the *CF*^*i*^ parameters namely X and Y positions, energy, and DOI limits. The mean values are calculated for all 24 detector and for all 121 calibration positions within each detector block. The error bars are calculated as the SD of all these 24 × 121 values. The *T2, T2B*, and *T2BB* cases are typically close to 1, meaning that they reflect well the ground truth. However, the *T1* and *T1B* cases are in general further from 1.

### Reconstructed Images

**Figure 5:**
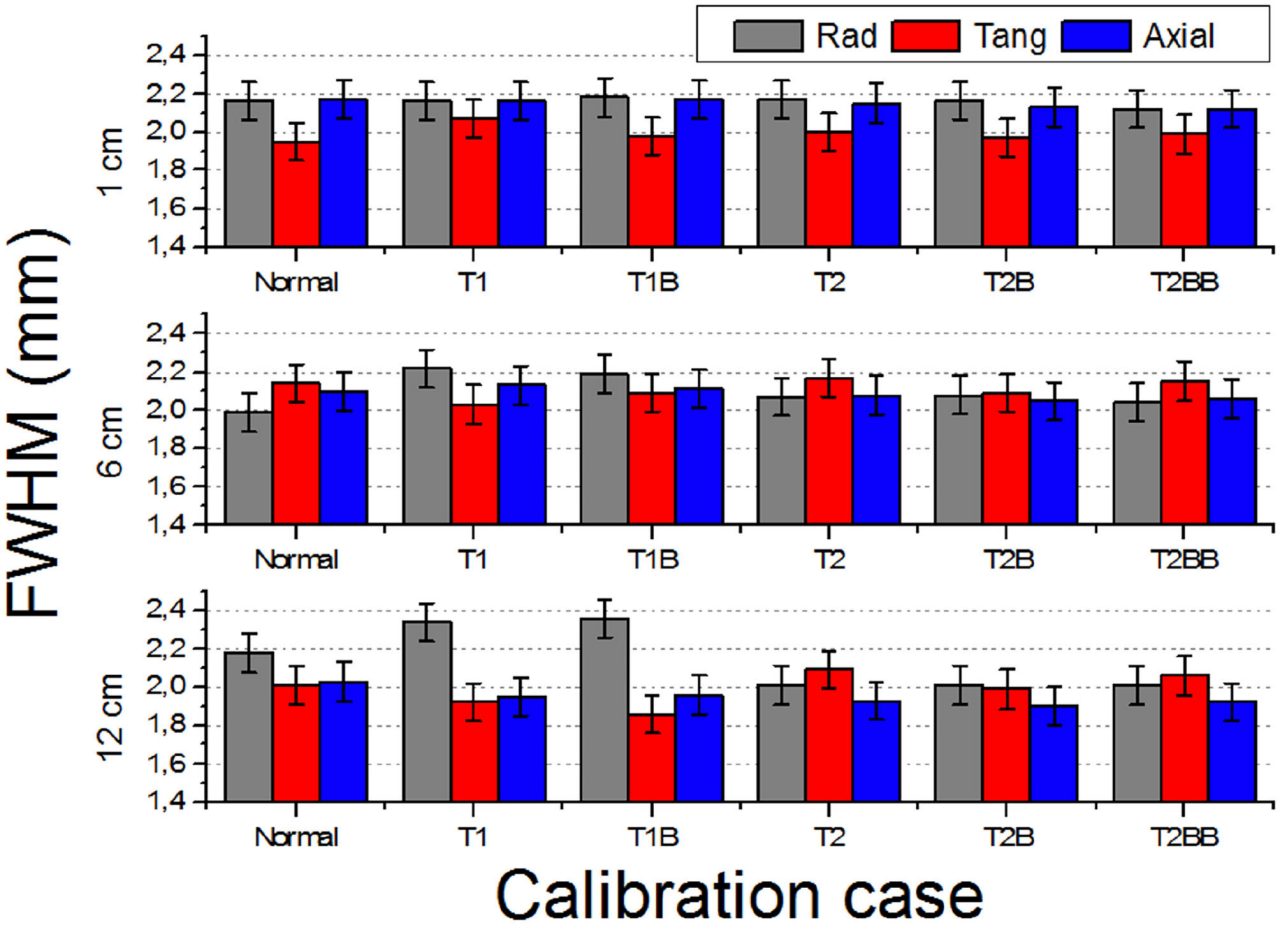
Reconstructed full width at half of the maximum (FWHM) (three components: radial, tangential, and axial) of the 1 mm in diameter source at off-radial positions 1, 6, and 12 cm.

Figure 5 depicts the FWHM values (radial, tangential, and axial) of the reconstructed images of the ^22^Na source versus the off-radial position. For the case closer to the center of the FOV (1 cm), all the cases exhibit very similar values. However, worse FWHM values are observed for the *T1* and *T1B* cases at radial positions far from the center, especially at the edges (12 cm) resulting in an elliptical shape of the sources.

**Figure 6:**
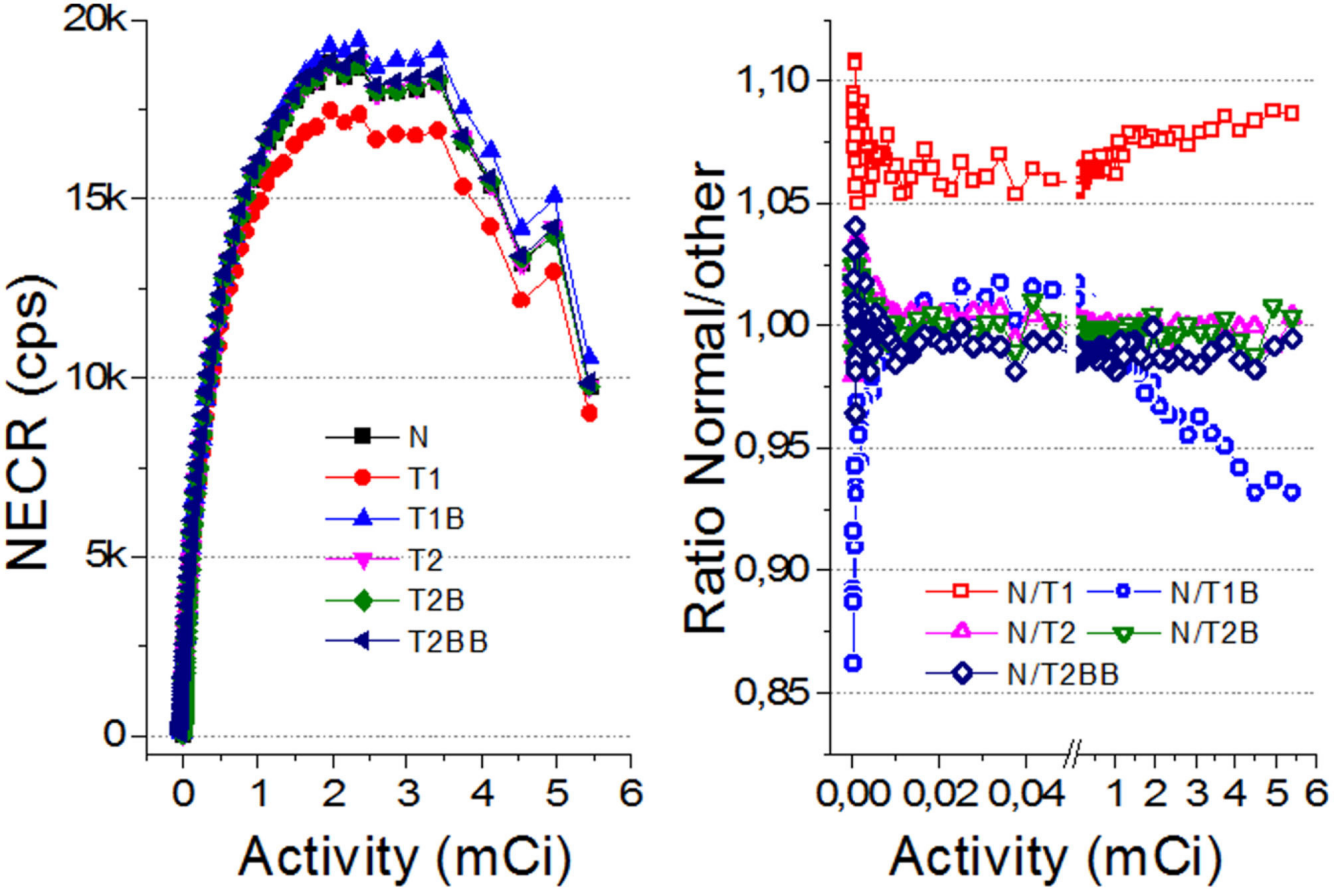
(Left) The noise equivalent count rate (NECR) curves for all the evaluated cases. (Right) Ratio of the NECR values for *Normal* with respect to all others. Notice there is a break between 0.5 and 0.6 mCi to expand the axis for lower values.

Figure 6 depicts the count rate capabilities of the system for each calibration method. In general, there is a better agreement for the *TEST2* approaches with respect to the *Normal* case. Some deviations are observed for the NECR curves regarding the *TEST1* cases (also for the True and Scatter/random ones but not shown here) at high activities. We have calculated the ratios of the NECR for the *Normal* case with respect to all others. The average ratio for the *T2, T2B*, and *T2BB* cases is as small as 0.2, 0.1, and 0.1%, respectively, with SDs of about 1% only. However, we found the ratios of 7 and 2% for the *T1* and *T1B*, respectively.

**Figure 7:**
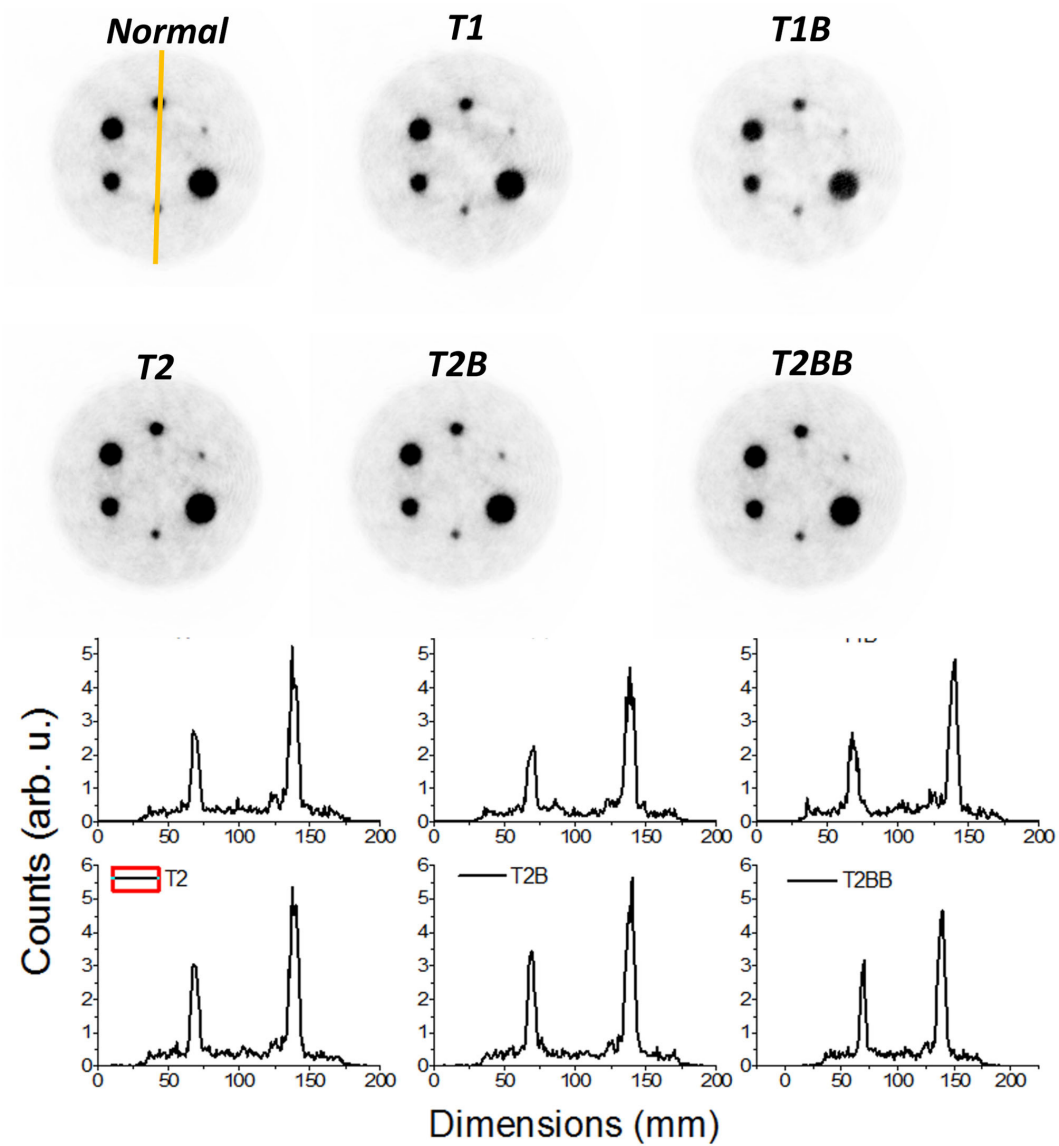
Top panels, reconstructed images of the image quality (IQ) phantom. Only 15% of the low color scale was used. Bottom panels, profiles along the smallest marked rods in the *Normal* case.

Figure 7 shows the reconstructed IQ phantom after applying the described calibration processes for all the cases. Qualitatively, the images and profiles are very similar. Slightly less uniform background is observed for the *TEST1* cases, as it can also be appreciated in the shown slice and projection at the bottom panels.

**Figure 8:**
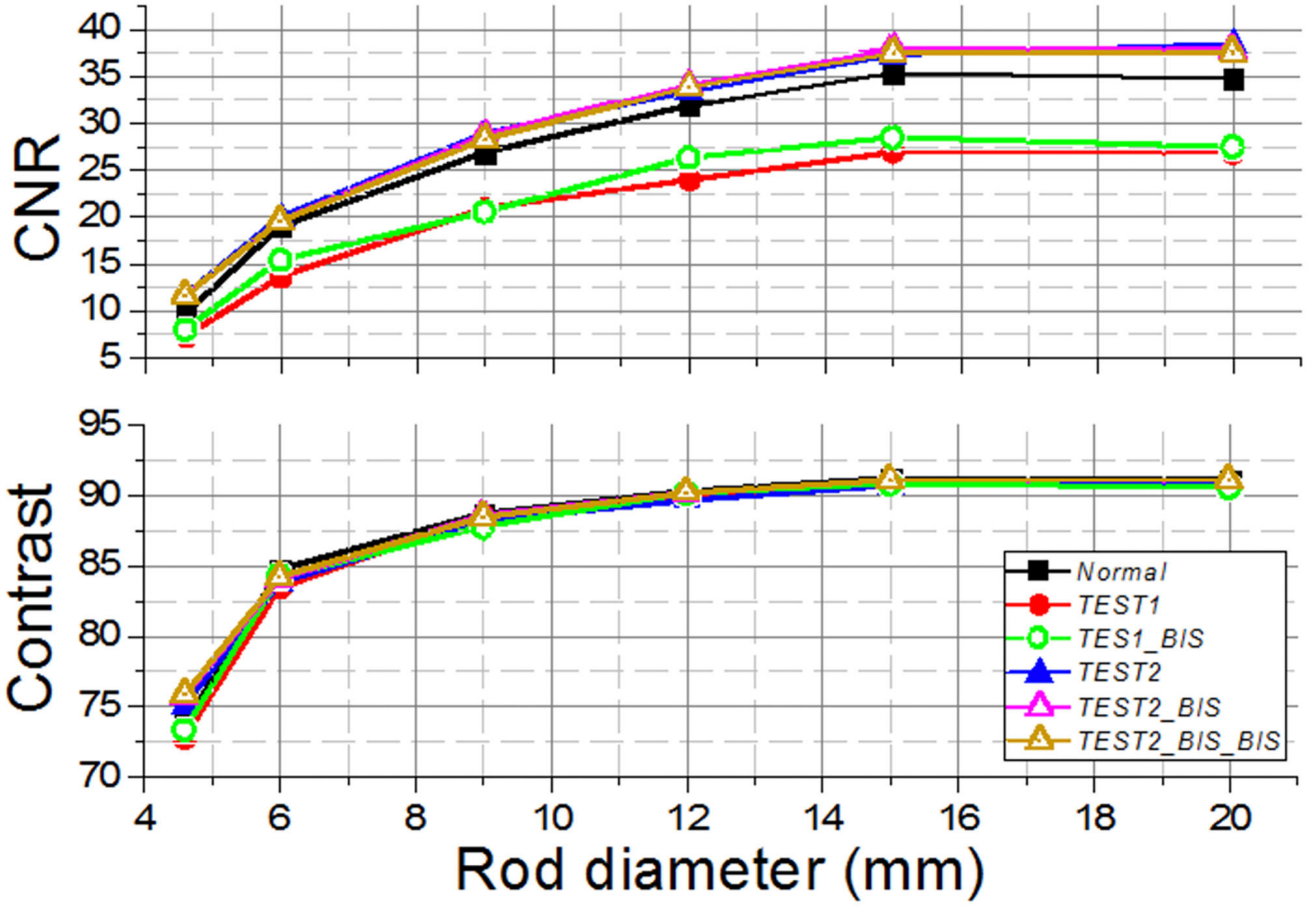
The contrast-to-noise ratio (CNR) (Top) and the contrast (Bottom) curves for the different *Normal, TEST1*, and *TEST2* cases acquired using the prostate dedicated PET system.

We observe the CNR values that are in general poor, most likely due to low acquisition times (Figure 8). Comparing the results obtained between *Normal* and the other methods, the CNR for *T1* and *T1B* are, on average, 28.5% lower. However, the *TEST2* cases exhibit similar values for the 4.5- and 6-mm rods, and better for the larger capillaries. An average improvement for all rods and tests of 8.4% is observed. We hypothesize that the improvement of CNR for the *TEST2* cases might be due to an improvement in the background uniformity caused by the averaging of three detector blocks.

## Discussion

In this work, we have studied the possibility to reduce the calibration time for monolithic-based PET systems. Different works are proposed to obtain reference dataset using the line sources and slit collimators or uncollimated sources without detector performance degradation, avoiding irradiating the crystal at a large number of known entry points across the entire surface, and thus, reducing the time calibration (6–11, 20, 22–24). Moreover, the use of simulated data for NN training or for LUT generation for ML position estimation (12, 13) allows for calibration time reduction. However, most of these methods demand higher computational requirements to be efficient.

In our approach, the calibration data are acquired using an array of collimated sources, instead of sequentially scanning individual radioactive sources across the crystal surface, which reduce the calibration times somewhat; however, in the *Normal* procedure each detector needs to be independently calibrated, which still leads to high time-consuming. Therefore, we have proposed two new calibration routines named *TEST1* and *TEST2* that reduce the calibration time from standard calibration of all 24 detectors of our prostate PET system (~72 h) to just 10 h in the case of *TEST2* and 3 h in the case of *TEST1* (as shown in Table 1). Notice that the times were estimated considering the activity of a source that can be typically found in the instrumentation laboratories and, therefore, higher activity sources would linearly improve the process. Using the high radioactivity sources and two screw bar and step motors would allow to create a robotic instrument to speed up the calibration acquisition and to prevent the radiation hazard at the same time. However, for the PET systems already installed in the research laboratories or clinical sites, introducing such a hardware setup is sometimes difficult.

**Table 1:** Estimation of calibration time processes for the different methods.

	**Steps/Tasks**				**Maximum calibration time**
	**Acquisitions**		**Computational time**		
	***11 × 11 ^**22**^Na sources array. (* **~** *10 μCi in total)***	***Uniformity***	***Shift map***	***LUT generation***	
**Normal**	24 detectors × (2–3 h/detector) ≈ 72 h	1 h	–	24 detectors × (1 min/detector) ≈ 20 min	72.3 h
**TEST1**	2–3 h	1 h	–	1 min	3 h
**TEST2**	3 detectors × (2–3 h/detector) ≈ 9 h	1 h	24 detectors × (24 sec/detector) ≈ 10 min	24 detectors × (1 min/detector) ≈ 20 min	10.5 h

An important implication of this reduction is that allow one to perform the calibration in one single working journey without the requirement of stopping, thus avoiding the additional complications. The uniform flood maps are obtained routinely during the PET calibration processes when for instance the normalization is performed. By reducing the calibration time without impacting the PET system performance, on the one hand, we are also minimizing the technical personnel exposure to radiation and, on the other hand, reducing the calibration cost associated to the supply of radioactive sources. An FDG dose used for calibration (370 MBq) costs approximately 275 € at our institution and lasts only for 1 day. Moreover, the proposed methodology simplifies the associated hardware, even if a low percentage of detectors are to be normally calibrated, such as in the *TEST2* (3/24 detectors), in comparison with calibrating all of them individually.

Our findings when comparing the results of the *TEST1* tests with the *Normal* case, showed some underperformance, as expected. Using one-detector calibration induces some errors due to many factors in the other 23 blocks, such as non-uniformities in the light collection, wrong coupling alignments of the photosensor and crystal, to name but a few. We observed that the reconstructed 1 mm sources show a worst performance for *T1* and *T1B* when they are far from the center FOV. Regarding the CNR, with the three different sets of detectors chosen for the *TEST2* cases, always a comparable performance to the *Normal* one case is found. Moreover, and somehow still to be understood, the CNR values outperformed those exhibited the *Normal* calibration. The *TEST1* cases are about 28% worst on average.

The *TEST2* methodology might be the key to exploit the use of large PET scanners based on the monolithic crystals because it has demonstrated the capabilities to significantly reduce the calibration times without system degradation, enabling to calibrate a system with very low computational cost and in a reasonable time-period in a clinical domain. For a system, such as the MINDView PET insert with 60 detectors blocks of 50 × 50 × 20 mm monolithic LYSO crystals (13), we struggled with a 10 days calibration process using the high activities of FDG sources, when calibrating 2–3 detectors simultaneously.

Obviously, the proposed methods require the detectors of each system to behave relatively similar, which is the case of commercially available PET scanners, since they go through the quality assessment tests during the manufacturing process. In our case, the assembly of all 24 detectors building the PET system was carried out following the same procedure, the readout electronics components have very small tolerances, and all the crystals and SiPM arrays are provided by the same manufacturer.

## Conclusion

We have proposed two new methodologies to reduce the calibration times for the monolithic-based PET systems and validated them using data acquired in a dedicated system for prostate imaging built of 24 monolithic crystals with 15 mm thickness each. The *TEST2* method, based on calibrating few detector blocks and then, making some fine tuning using the uniform calibration maps (routinely obtained when the corrections based on uniform radiation are applied), has shown the possibility of simplifying and accelerating the calibration process without system performance degradation and without high computational cost. Therefore, this proposed method allows to solve one of the obstacles to translate to the clinics large monolithic-based PET scanners.

## Data Availability Statement

The raw data supporting the conclusions of this article will be made available by the authors, without undue reservation.

## Author Contributions

MF has designed the experiments, the calibration of all detectors, and elaborated the draft manuscript. SE has analyzed part of the data. GC has taken care of the normalization correction and reconstruction of the data. AG-M has supervised the calibration processes and conducted the experimental data acquisitions. AG has managed the different contributions, wrote the final manuscript, and interpreted the results. All authors contributed to the article and approved the submitted version.

## Funding

This work was supported in part by the Spanish Ministerio de Ciencia e Innovacion under Grant No. PID2019-107790RB-C21 and co-funded by the European Union ERDF funds (European Regional Development Fund [ERDF]) of the Comunitat Valenciana 2014-2020, with reference IDIFEDER/2018/032 (High-Performance Algorithms for the Modeling, Simulation, and early Detection of diseases in Personalized Medicine). This work was in part also supported by the Imagen Molecular de Alta Sensibilidad (IMAS) project launched by the Conselleria de Sanitat Universal i Salut Publica of the Goverment of Valencia Region, announced in the BOE 328, December 28, 2020, co-funded at 50% by the ERDF. AG-M was supported by Valid Program for Researchers in Postdoctoral Phase of the Ministry of Labor and Social Economy (Generalitat de Valencia) and the EU Social Fund. MF was supported by the Program for Researchers in Predoctoral Phase of the Ministry of Labor and Social Economy (Generalitat de Valencia) and the EU Social Fund.

## Conflict of Interest

The authors declare that the research was conducted in the absence of any commercial or financial relationships that could be construed as a potential conflict of interest.

## Publisher's Note

All claims expressed in this article are solely those of the authors and do not necessarily represent those of their affiliated organizations, or those of the publisher, the editors and the reviewers. Any product that may be evaluated in this article, or claim that may be made by its manufacturer, is not guaranteed or endorsed by the publisher.
